# Low Dose Rapamycin Exacerbates Autoimmune Experimental Uveitis

**DOI:** 10.1371/journal.pone.0036589

**Published:** 2012-05-04

**Authors:** Zili Zhang, Xiumei Wu, Jie Duan, David Hinrichs, Keith Wegmann, Gary L. Zhang, Mark Hall, James T. Rosenbaum

**Affiliations:** 1 Department of Pediatrics, Oregon Health & Science University, Portland, Oregon, United States of America; 2 Portland VA Medical Center, Portland, Oregon, United States of America; 3 Department of Ophthalmology, Oregon Health & Science University, Portland, Oregon, United States of America; 4 Department of Medicine, Oregon Health & Science University, Portland, Oregon, United States of America; La Jolla Institute for Allergy and Immunology, United States of America

## Abstract

**Background:**

Rapamycin, a potent immune modulator, is used to treat transplant rejection and some autoimmune diseases. Uveitis is a potentially severe inflammatory eye disease, and 2 clinical trials of treating uveitis with rapamycin are under way. Unexpectedly, recent research has demonstrated that low dose rapamycin enhances the memory T cell population and function. However, it is unclear how low dose rapamycin influences the immune response in the setting of uveitis.

**Design and Methods:**

B10.RIII mice were immunized to induce experimental autoimmune uveitis (EAU). Ocular inflammation of control and rapamycin-treated mice was compared based on histological change. ELISPOT and T cell proliferation assays were performed to assess splenocyte response to ocular antigen. In addition, we examined the effect of rapamycin on activation-induced cell death (AICD) using the MitoCapture assay and Annexin V staining.

**Results:**

Administration of low dose rapamycin exacerbated EAU, whereas treating mice with high dose rapamycin attenuated ocular inflammation. The progression of EAU by low dose rapamycin coincided with the increased frequency of antigen-reactive lymphocytes. Lastly, fewer rapamycin-treated T cells underwent AICD, which might contribute to exaggerated ocular inflammation and the uveitogenic immune response.

**Conclusion:**

These data reveal a paradoxical role for rapamycin in uveitis in a dose-dependent manner. This study has a potentially important clinical implication as rapamycin might cause unwanted consequences dependent on dosing and pharmacokinetics. Thus, more research is needed to further define the mechanism by which low dose rapamycin augments the immune response.

## Introduction

Rapamycin (Sirolimus) is a macrolide initially discovered in bacterium *Streptomyces Hygroscopicu* [1]. Through FK binding protein 12, rapamycin interacts with mammalian target of rapamycin (mTOR), a serine/threonine protein kinase critical for cell metabolism and proliferation [2]. Early studies showed that rapamycin exerts a potent immunosuppressive effect [1], [3]. It inhibits the lymphocyte response to IL-2, and promotes regulatory T cell (Treg) development and function [1], [3]. As a result, rapamycin, either alone or in conjunction with other immunosuppressants, has become a primary immunomodulatory reagent for preventing transplant organ rejection. Furthermore, rapamycin has been employed to treat a number of autoimmune/inflammatory diseases [4]–[6]. In addition, its anti-proliferative effect makes rapamycin a promising agent for suppressing tumor growth and coronary arterial restenosis [7]–[9]. Thus, rapamycin exerts its therapeutic impact on a broad range of clinical conditions. Despite the fact that rapamycin is an immunosuppressant, some patients treated with this medication develop idiosyncratic interstitial pneumonitis [10]–[12]. Moreover, recent studies have demonstrated that low dose rapamycin treatment enhances memory T cell development and function in a murine viral infection model [13]. This effect appears to be mediated by mTOR [13], which was supposedly inhibited by rapamycin. This important finding is unexpected and bears an important clinical implication. For instance, low dose rapamycin could be used as an adjuvant to improve vaccine efficacy and to augment memory response to infection and cancer therapy. Most autoimmune diseases exhibit a chronic and relapsing course, which is mediated by effector and memory lymphocytes. Thus, suboptimal dosing of rapamycin might exacerbate autoimmune and inflammatory diseases. This may also explain the rare but severe pulmonary complication of rapamycin in some patients.

Autoimmune uveitis is a potentially serious ophthalmologic disorder characterized by intraocular inflammation. It is commonly associated with many systemic diseases characterized by a dysregulated immune response (e.g. sarcoidosis, ankylosing spondylitis, inflammatory bowel disease). T lymphocytes play an important role in the pathogenesis of many forms of uveitis by recognizing antigen and orchestrating the immune response [13], [14]. Recently, 2 clinical trials of intraocular rapamycin treatment for active uveitis (NCT00908466 and NCT00876434) were begun. In light of the recent “paradoxical” effect of low dose rapamycin on T cells, a pre-clinical study is critically needed to assess the impact of low dose rapamycin on the outcome of uveitis. Such study could provide valuable information on the dosing of rapamycin for treating uveitis and other immune-related diseases. Moreover, it will advance our knowledge of the multifaceted effects of rapamycin on T cell-mediated immune responses.

In this study, we have demonstrated that administration of low dose rapamycin exacerbated and prolonged experimental autoimmune uveitis (EAU), whereas treating mice with a high dose of rapamycin attenuated ocular inflammation. In addition, the treatment with low dose rapamycin expanded the peripheral antigen-reactive-T cell population. Furthermore, rapamycin treatment reduced activation-induced cell death in the lymphocytes. Thus, these findings suggest that low dose rapamycin amplifies and prolongs the T cell response, thereby leading to more severe uveitis. Although the mechanism by which low dose rapamycin augments inflammation remains to be fully defined, the significance of this study is apparent. Rapamycin therapy might cause unwanted consequences due to the dosing regimen and individual pharmacokinetic variations.

## Materials and Methods

### Mice

Six-week-old female B10.RIII mice (Jackson Laboratory, Bar Harbor, ME) were used for the experiments. The animal experimental protocols have been approved by the institutional animal care and use committee at Oregon Health & Science University (IACUC number ISO1307).

### Experimental autoimmune uveitis

EAU was induced in B10.RIII mice by subcutaneous immunization (near the base of the tail) with 50 µg interphotoreceptor retinoid-binding protein peptide 161–180 (IRBP_161–180_) (Ser-Gly-Ile-Pro-Tyr-Ile-Ile-Ser-Tyr-Leu-His-Pro-Gly-Asn-Thr-Ile-Leu-His-Val-Asp) (AnaSpec, Fremont, CA) in 200 µl complete Freund's adjuvant (Sigma-Aldrich, St. Louis, MO). On days 14 and 21 after immunization, the eyes were harvested for histology and real time-PCR.

### Histology

For histological evaluation, the eyes were fixed in 3% paraformaldehyde. Then, the tissues were embedded in paraffin, sectioned, and stained with hematoxylin and eosin (H&E). Ocular inflammation was assessed by light microscopy, and the severity of EAU was graded on a four-point scale based on inflammatory cell infiltration, retinal folding and destruction [15].

### Real time-PCR and Mouse Apoptosis PCRarray

Total RNA from whole eye tissue was isolated with RNAeasy Mini Kit (Qiagen, Valencia, CA). First-strand cDNA synthesis was accomplished with oligo (dT)-primed Omniscript reverse transcriptase kit (Qiagen). Gene-specific cDNA was amplified by PCR using mouse specific primer pairs (IFN-γ sense: 5′-GCT TTG CAG CTC TTC CTC AT-3′ and IFN-γ anti-sense: 5′-GTC ACC ATC CTT TTG CCA GT-3′; IL-17A sense: 5′-GTG GCG GCT ACA GTG AAG GCA-3′, and IL-17A antisense: 5′-GAC AAT CGA GGC CAC GCA GGT-3′; HIF-α1 sense: 5′-TCC ATG TGA CCA TGA GGA AA-3′ and HIF-α1 anti-sense: 5′-CTT CCA CGT TGC TGA CTT GA-3′; VEGF sense: 5′-AGC ACA GCA GAT GTG AAT GC-3′ and VEGF anti-sense: 5′-AAT GCT TTC TCC GCT CTG AA-3′; Foxp3 sense: 5′-CCC CTA GTT CCA ACC TAG CC-3′, and Foxp3 antisense: 5′-TAC CAA GGC AGG CTC TTC AT-3′; IL-10 sense: 5′-CCA AGC CTT ATC GGA AAT GA-3′, and IL-10 antisense: 5′-TTT TCA CAG GGG AGA AAT CG-3′; TGF-β1 sense: 5′-TGG AGC AAC ATG TGG AAC TC-3′, and TGF-β1 antisense: 5′-AGC CCT GTA TTC CGT CTC CT-3′; β-actin sense, 5′-ATG CCA ACA CAG TGC TGT CT-3′, and β-actin antisense, 5′-AAG CAC TTG CGG TGC ACG AT- 3′). The real-time PCR was performed using a RT^2^ Realtime PCR Master mix (SABiosciences, Valencia, CA), and running for 40 cycles at 95°C for 15 sec and 55°C for 40 sec. The mRNA levels of investigated genes in each sample were normalized to β-actin mRNA and quantified using a formula 2^(−ΔΔCt)^. The result was expressed as fold change in the groups treated with rapamycin compared to control EAU.

RNA from cultured DO11.10 splenocytes was isolated to detect the effect of rapamycin on apoptosis-related gene transcription. Mouse Apoptosis real-time PCR Array was performed independently 3 times according to the manufacturer's protocol (SABiosciences), and GEArray® Expression Analysis Suite software was used for data extraction and analysis.

### Cell culture, isolation, and stimulation

The spleens were harvested from B10.RIII or DO11.10 mice, and single cell suspension was prepared by passing the tissue through a 70 µm cell strainer (BD Biosciences, Mountain View, CA). Red blood cells (RBC) were lysed with 1× RBC lysis buffer (Sigma, St Louis, MO) at room temperature for 5 min. The spleen cell suspension was washed with RPMI 1640, and then cultured in RPMI 1640 with 10% fetal bovine serum (FBS) in an atmosphere of 95% air and 5% CO_2_ at 37°C with 4 µg/ml IRBP_161–180_ peptide for 72 hours.

### Flow cytometry

B10.RIII or DO11.10 splenocytes were suspended in phosphate buffered saline (PBS) containing 2% FBS. Anti-CD4, anti-CD8, anti-CD44, and anti-CD62L antibodies conjugated with different fluorescent colors (eBioscience, San Diego, CA) were used to label these cell surface markers for 60 minute on ice. For Foxp3 staining, the cells were fixed and permeabilized overnight with 1× fixation/permeabilization solution (eBioscience) at 4°C. Then these cells were stained intracellularly with monoclonal antibody against Foxp3 (eBioscience) for 1 hour at 4°C. Data acquisition was performed on a FACSCalibur flow cytometer, and data were analyzed using CellQuest software. To assess apoptosis, the cells were incubated with Annexin V (eBioscience) and analyzed by flow cytometry.

### T cell proliferation assay

T cell proliferation responses were assessed by plating 5×10^5^/200 µl splenocytes from IRBP_161–180_-immunized B10.RIII mice in triplicate into a 96-well flat-bottom tissue culture plate. IRBP_161–180_ (0.4 µg/200 µl) or media control were added in a volume of 10 µl/well. Cultures were incubated for 72 h at 37°C in 7% CO_2_. Wells were pulsed for the final 18 h with 0.5 µCi per well [^3^H]thymidine. The cells were harvested onto glass fiber filters, and [^3^H]thymidine uptake was measured using a liquid scintillation counter (1205 Betaplate:Wallac, Turku, Finland). Stimulation index was calculated by dividing the cmp of sample well by the blank well.

### ELISPOT assay and ELISA

The production of interferon (IFN)-γ and interleukin (IL)-17 was quantitated by the ELISPOT assay. Isolated cells were washed once and seeded at a density of 3×10^5^ cells per well in triplicate into 96-well sterile 0.45 µm MultiScreen-HA filter plates (Millipore, Billerica, MA) coated with 5 µg/mL anti-IFN-**γ** or anti-IL-17 monoclonal antibody (eBioscience). These cells were incubated in the presence or absence of IRBP (4 µg/ml) for 24 hours at 37°C in 5% CO_2_. Then the cells were washed, IFN- **γ**- and IL-17-producing cells were detected as positive spots by addition of a second biotin-conjugated anti-IFN- **γ** or anti-IL-17 antibody (eBioscience), followed by streptavidin alkaline phosphatase and 5-bromo-4-chloro-3-indolyl phosphate toluidine p-nitro blue tetrazolium chloride substrate (KPL, Gaithersburg, MD). Spots were analyzed by an AID Elispot high resolution reader system EliSpot 04 HR (AID Elispot, Strassberg, Germany).

Fifty µl culture media of isolated B10.RIII splenocytes from various experimental groups were collected for ELISA to measure the IFN-γ, IL-10, and IL-17 levels according to the manufacturer's protocols (BioLegend, San Diego, CA).

### MitoCapture apoptosis assay

Apoptosis was assessed using the MitoCapture mitochondrial apoptosis detection kit (Biovision, Mountain View, CA) according to the manufacturer's instructions. Splenocytes from DO11.10 were cultured with and without 100 nM rapamycin for 48 hours. Then, apoptosis was induced by stimulating the cells with ovalbumin (OVA) for 3 days. Then, these cells were incubated in MitoCapture solution at 37°C in a 5% CO_2_ incubator for 20 min. The splenocytes were washed with PBS and analyzed by confocal microscopy.

### Statistics

Data are expressed as the average ± SEM. For EAU scoring, median difference between control and experimental groups was compared using Mann-Whitney U test. Other statistical probabilities were evaluated by Student's *t* test or ANOVA, with a value of *p*<0.05 considered significant.

## Results

### Exacerbation of experimental autoimmune uveitis by low dose but not high dose rapamycin

**Figure 1:**
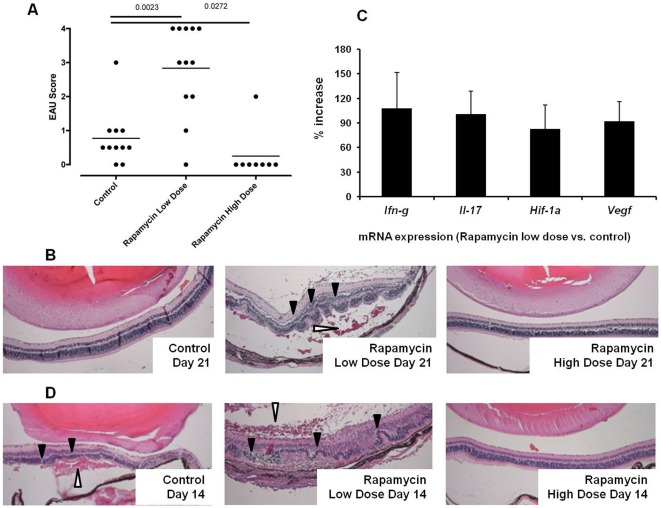
Low dose rapamycin but not high dose rapamycin exacerbates IRBP-induced EAU in B10.RIII mice. The mice received daily rapamycin (1.5 or 7.5 µg per mouse) via intraperitoneal injection. On day 21, eyes were harvested for histological EAU scoring (A) (Each dot represents one eye from each mouse in different experimental groups); Representative histology of severe retinal inflammation, folding and damage in low dose rapamycin-treated EAU (B); Ocular expression of *Ifn-γ, Il-17, Hif-1α,* and *Vegf* transcripts was measured by quantitative PCR. The mRNA level was normalized to β-actin, and the relative quantity was further compared between control and low dose rapamycin-treated groups (C) (The data represent the mean of 6 individual animals in each group). Some mice were euthanized on day 14 after IRBP_161–180_ inoculation, and ocular inflammation was compared histologically between control EAU and rapamycin-treated groups (D) (Filled arrow heads: retinal folding; Open arrow heads: peri-retinal hemorrhage) (Representative histology image of 4 mice in each experimental group).

To assess the effect of rapamycin on EAU, we treated B10.RIII mice with intraperitoneal injection of 1.5 or 7.5 µg rapamycin daily after IRBP_161–180_ sensitization. On day 21, EAU was scored based upon inflammatory cell infiltration, vasculitis, retinal folding, and destruction. Compared to control EAU, the daily administration of 1.5 µg rapamycin significantly augmented the severity of EAU, whereas the treatment with 7.5 µg rapamycin virtually inhibited IRBP_161–180_-induced uveitis (P<0.05) (Fig. 1A). As illustrated in Fig. 1B, the mice in the low dose rapamycin-treated group consistently exhibited more severe retinal destruction, marked retro-retinal hemorrhage and retinal detachment. In addition, we examined the expression of mRNA for the inflammatory cytokines IFN-γ and IL-17 in the eyes of these animals. Total RNA from whole eye was isolated on day 21 after EAU induction. In line with the histological change, real time-PCR revealed an increase of *Ifn-γ* and *Il-17* transcripts in the group that received low dose rapamycin compared to the control group with EAU (Fig. 1C). Hypoxia-inducible factor-1α (HIF-1α) and vascular endothelial growth factor (VEGF) are implicated in inflammation and ocular pathology including uveitis [16]–[20]. Furthermore, recent studies have shown that mTOR signaling regulates HIF-1α and VEGF expression [21]–[23]. Low dose rapamycin can potentially impact on ocular vasculature during uveitis by altering HIF-1α and VEGF expression. This could contribute to the retinal hemorrhages and detachment. Thus, we examined the ocular transcription of HIF-1α and VEGF. Compared to the control EAU group, real time-PCR showed that low dose rapamycin treatment increased ocular *Hif-1α* and *Vegf* mRNA expression during EAU (Fig. 1C).

Recently, similar to the observations of others, we reported that the ocular inflammation peaks at day 14 and subsides at day 21 after IRBP sensitization in our EAU model [24]. Thus, the uveitis seen in the low dose rapamycin-treated group on day 21 could be simply due to delayed onset of EAU. To rule out this possibility, we compared the severity of EAU between the groups with and without low dose rapamycin on day 14. As shown in Fig. 1D, the ocular inflammation and retinal injury are comparable in both control and rapamycin-treated groups. This result indicates that low dose rapamycin prolongs and exacerbates EAU instead of merely shifting the kinetics of the ocular immune response.

These findings clearly suggest that low dose rapamycin up-regulates the immune response in the setting of T cell-mediated ocular inflammation. The dose-dependent paradoxical effect of rapamycin on EAU is novel, and it carries potentially significant implications as low dose rapamycin might cause a detrimental consequence if the original therapeutic intention is to suppress immune response.

### The effect of low dose rapamycin during the different stages of EAU development

**Figure 2:**
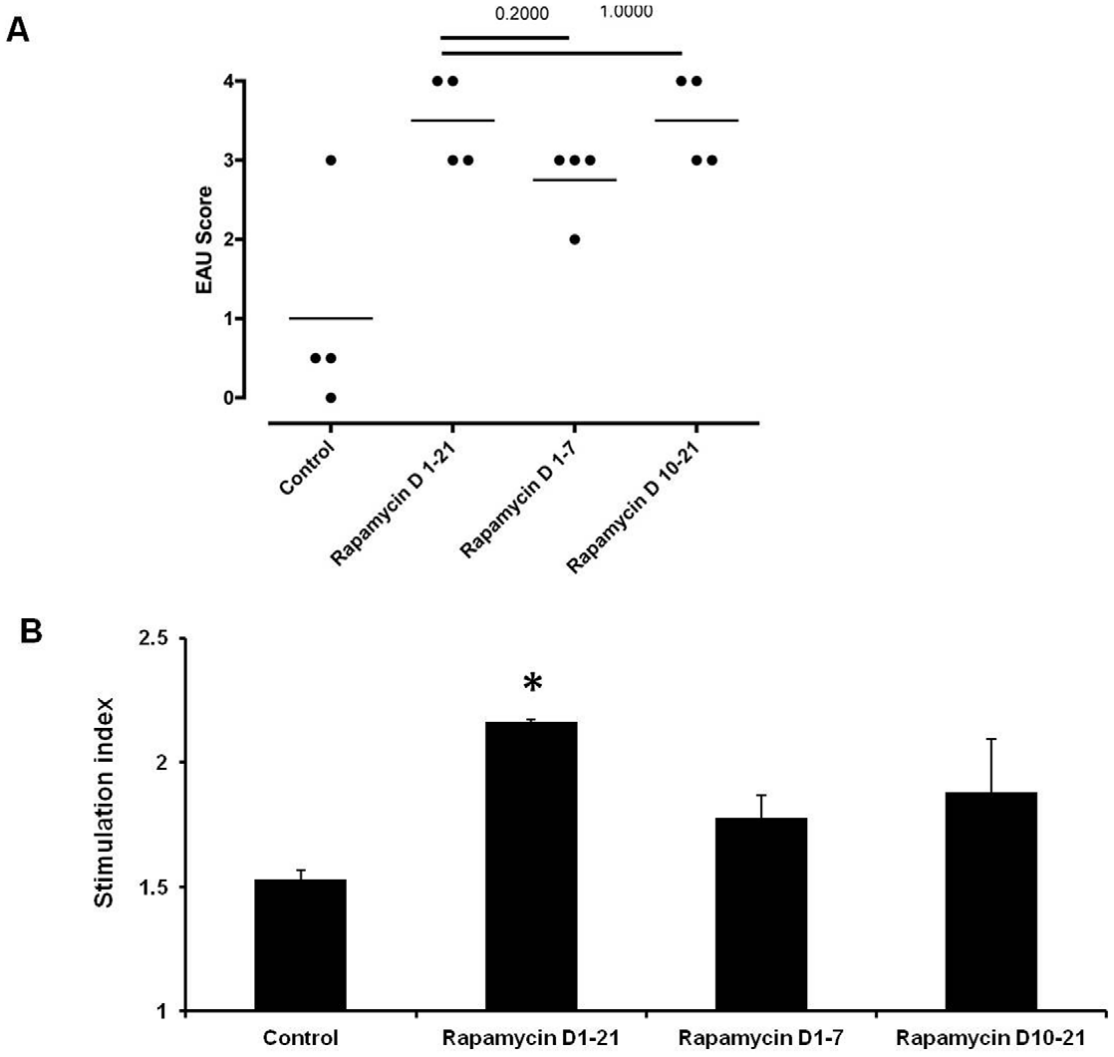
Treatment of low dose rapamycin during IRBP sensitization subsequently causes severe EAU. Rapamycin (1.5 µg per mouse) was administered intraperitoneally during IRBP_161–180_ sensitization (days 0–7) or after EAU onset (days 10–21) after IRBP_161–180_ immunization. On day 21, the eyes were harvested for histological evaluation and EAU scoring (A) (Each dot represents one eye from each mouse in different experimental groups); T cell proliferation assay of the lymphocytes from control and rapamycin-treated mice in response to 72-hour *in vitro* IRBP_161–180_ stimulation (The data represent the mean of 6 individual animals in each group; * p<0.05).

Recently, Araki et al. reported that treatment of mice with low dose rapamycin promotes the development of virus-specific T cells in a lymphocytic choriomeningitis virus infection model [13]. Thus, it is feasible to postulate that low dose rapamycin could augment the ocular inflammation by up-regulating uveitogenic T cells. During the first week of antigen encounter, naive T cells are activated and differentiated to effector lymphocytes. To further investigate the effect of low dose rapamycin on initial uveitogenic T cell development, we treated mice intraperitoneally with 1.5 µg rapamycin for the first 7 days following IRBP inoculation. In addition, another group of mice received daily rapamycin from days 10–21 when uveitis becomes histologically apparent. This enabled us to evaluate the impact of low dose rapamycin on T cell activation and subsequent T cell response. On day 21 after IRBP sensitization, the ocular inflammation was mild in the control EAU group with an average EAU score of 1, whereas the EAU score increased to 3.5 after the mice were treated with rapamycin from days 1–21. In addition, the mice receiving rapamycin during IRBP immunization phase (days 1–7) or after disease onset (days 10–12) developed a mean EAU score of 2.75 and 3.5, respectively (Fig. 2A). This suggests that low dose rapamycin impacts the antigen sensitization process and amplifies uveitogenic T cell activation. To further test if low dose rapamycin enhances the ocular antigen-specific T cell response, we harvested splenocytes from these mice on day 21 and re-stimulated the cells with IRBP_161–180_ *in vitro*. The lymphocytes from low dose rapamycin-treated groups exhibited a higher cell proliferation in response to IRBP than the control group (Fig. 2B). Among the animals receiving rapamycin at different stages of EAU development, the mice displayed the most robust proliferative response to the antigen when they were treated with rapamycin through the entire course of the experiment (days 1–21). This indicates that low dose rapamycin exacerbates EAU by a combined effect on eliciting and propagating T cell response.

### Potential mechanism mediated by low dose rapamycin

**Figure 3:**
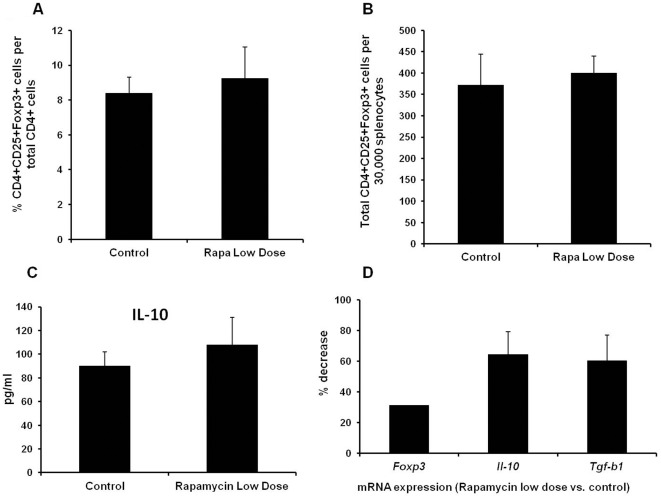
Low dose rapamycin treatment does not affect peripheral CD4+CD25+Foxp3+ Treg cells but attenuates ocular transcription of Foxp3, IL-10, and TGF-β. EAU and daily treatment of rapamycin (1.5 µg/mouse) are described previously. On day 21, the spleens were harvested, and peripheral Treg cells were assessed by flow cytometry for CD4, CD25, and Foxp3 staining. The percentage and total number of CD4+CD25+Foxp3+ Treg cells were compared between control EAU and low dose rapamycin-treated groups (A and B). The harvested splenocytes (2.5×10^6^/ml) were further stimulated with IRBP_161–180_ (4 µg/ml) *in vitro* for 72 hours. The cell culture media were collected for ELISA of IL-10 (C). In addition, the eyes were harvested, and ocular total RNA was isolated for real time-PCR analysis of Foxp3, IL-10, and TGF-β expression. The level of investigated mRNA was normalized to β-actin, and the relative quantity was further compared with EAU group without low dose rapamycin treatment (All the data represent the mean of 6 animals per group).

Previously, we reported that EAU subsided by day 21 [24]. Here, we found that low dose rapamycin prolonged the course of uveitis. The continuing ocular inflammation could be due to suppression of immune regulation or extension of effector T cell life span. Thus, we went further to investigate the potential mechanism by which low dose rapamycin augments EAU. Since recent studies have shown that mTOR is implicated in Foxp3+ Treg differentiation [25]–[27], we first examined the impact of low dose rapamycin on the Treg in EAU. On day 21 after IRBP_161–180_ priming, CD4+CD25+Foxp3+ T cells in the spleen were analyzed by flow cytometry. As illustrated in Figs. 3A and 3B, there was no significant change of peripheral Treg population in the EAU mice treated with and without low dose rapamycin. Furthermore, we stimulated the splenocytes of these mice with IRBP_161–180_ *in vitro* for 72 hours. IL-10 level in the culture media was determined by ELISA. Compared to control EAU group, low dose rapamycin did not significantly alter the production of IL-10 in the animals that received daily low dose rapamycin *in vivo* (Fig. 3C). To assess the impact of rapamycin on ocular Treg cells, Foxp3, IL-10, and TGF-β expression in the eye was measured by RT-PCR. Compared to control EAU, ocular transcription of *Foxp3*, *Il-10*, and *Tgf-β* was attenuated in the low dose rapamycin-treated group (Fig. 3D). This suggests that low dose rapamycin may inhibit the ocular trafficking of Treg cells. Alternatively, severe local inflammation could suppress the expression of Foxp3, IL-10, and TGF-b in Treg cells.

**Figure 4:**
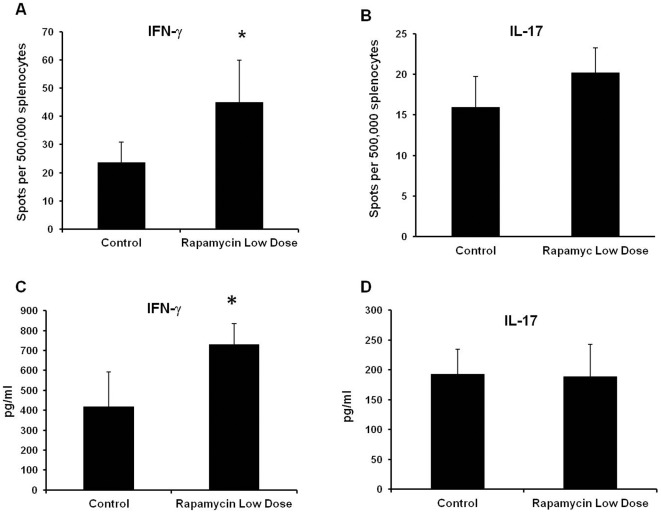
Daily low dose rapamycin treatment increases IRBP-reactive T cells in EAU mice. EAU induction and rapamycin treatment are described previously. On day 21, the splenocytes were harvested, and further stimulated with IRBP_161–180_ (4 µg/ml) *in vitro* for 24 hours. Then, the frequency of IFN-γ and IL-17-producing cells was measured by ELISPOT (A and B). In addition, the harvested splenocytes (2.5×10^6^/ml) were further stimulated with IRBP_161–180_ (4 µg/ml) *in vitro*. The cell culture media were collected for ELISA of IFN-γ (C) and IL-17 (D) (All the data represent the mean of 5–7 animals per group; * p<0.05).

**Figure 5:**
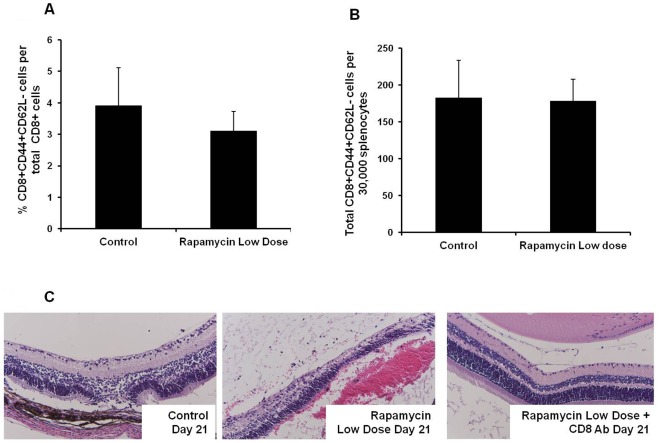
Low dose rapamycin treatment does not affect the number of peripheral activated CD8+ cells. However, anti-CD8 antibody attenuates rapamycin-enhanced EAU. EAU and daily treatment of rapamycin (1.5 µg/mouse) are described previously. On day 21, the spleens were harvested, and activated CD8+ cells were assessed by flow cytometry for the surface expression of CD44 and CD62L. The percentage and total number of CD8+CD44+CD62L− cells were compared between control EAU and low dose rapamycin-treated groups (A and B) (The data represent the mean of 5–7 animals per group). Some B10.RIII mice received 200 µg of anti-CD8 antibody intraperitoneally on days 1 and 8 to deplete peripheral CD8+ cells. On day 21, EAU was evaluated by histology (C) (Representative histology image of 3–4 mice in each experimental group).

Several recent studies demonstrated that rapamycin promotes an antigen-specific T cell response [13], [28]–[30]. Therefore, we postulated that low dose rapamycin affects the activated T cell population, thereby contributing to the exacerbation of EAU. To test this hypothesis, we compared the frequency of peripheral IRBP-reactive lymphocytes between control EAU and in groups treated with 1.5 µg rapamycin from days 1–21. On day 21 after EAU induction, splenic lymphocytes were stimulated with IRBP_161–180_ *in vitro*, and the number of IFN-γ- and IL-17-producing cells was analyzed by ELISPOT assay. Compared to the control EAU group, low dose rapamycin administration resulted in a significant increase of the relative number of lymphocytes that produced IFN-γ in specific response to IRBP_161–180_ (Fig. 4A). However, there was no marked change of IL-17-producing cells between control and rapamycin-treated groups (Fig. 4B). We also measured secreted IFN-γ and IL-17 in the culture media. Consistent with the ELISPOT result, ELISA showed that low dose rapamycin treatment significantly increased IFN-γ but not IL-17 production (Figs. 4C and 4D). This suggests that low dose rapamycin does not enhance peripheral Th17 differentiation in this model. During ocular inflammation, both activated Th1 and Th17 cells traffic to and concentrate in the eye. This could explain the increase of ocular IFN-γ and IL-17 transcription in the rapamycin-treated group (Fig. 1C). Although the induction of EAU is largely elicited by CD4+ T cells, IRBP_161–180_ has been shown to induce a CD8+ T cell response [31]. Recently, several studies have demonstrated that low-dose rapamycin up-regulates CD8+ T cells [13], [30]. Therefore, we investigated if low dose rapamycin could increase activated CD8+ T cells, thereby resulting in exaggerated ocular inflammation. On day 21 after IRBP_161–180_ priming, T cell activation markers CD44 and CD62L of splenic CD8+ lymphocytes were analyzed by flow cytometry in the mice treated with and without rapamycin. As illustrated in Figs. 5A and 5B, low dose rapamycin did not significantly increase the number of CD8+CD44+CD62L− population. To further examine the impact of low dose rapamycin on CD8+ cell function, we investigated if depletion of CD8+ T cells attenuated rapamycin-enhanced EAU. Two hundred µg anti-mouse CD8 monoclonal antibody (Clone 2.43) (BioXcell, Lebanon, NH) was administered intraperitoneally on days 1 and 8 upon the induction of EAU. On day 21, ocular inflammation was assessed by intravital microscopy. Compared to the control EAU group, low dose rapamycin treatment exacerbated ocular inflammation and retinal damage (Fig. 5C). However, anti-CD8 monoclonal antibody significantly reduced the severity of EAU in rapamycin-treated mice (Fig. 5C). This suggests that CD8+ T cells contribute to low dose rapamycin-enhanced ocular immune response, even though low dose rapamycin does not increase the percentage of CD8 cells in the spleen.

**Figure 6:**
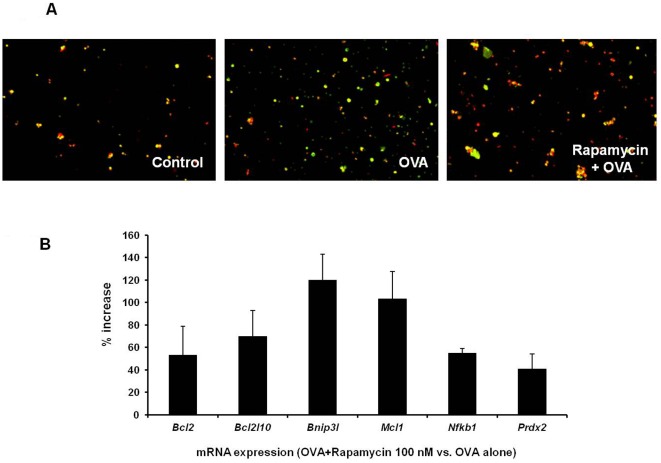
Pre-treatment of low dose rapamycin attenuates activation-induced cell death of DO11.10 CD4+ T cells. Splenocytes were harvested from DO11.10 mice. They were pre-treated with or without 100 nM rapamycin for 48 hours. Then, these cells were cultured in the presence of OVA for additional 72 hours. Apoptosis of DO11.10 splenocytes was detected by MitoCapture assay (Representative image of the cells from 3 individual mice in each group) (A). In addition, 24 hours after OVA stimulation, total RNA was collected from cultured DO11.10 splenocytes, and Mouse Apoptosis PCRarray was performed to compare the change of anti-apoptotic gene expression between control and rapamycin treated cells (The data represent the mean of 3 independent experiments with total 3 mice in each group).

When peripheral T cells are activated by a specific antigen, they rapidly enter clonal expansion. Then, a majority of T cells undergo activation-induced cell death (AICD), a critical process for containing the immune response [32]–[35]. Deletion of the apoptotic molecules regulating AICD leads to exaggerated inflammation [36], [37]. A recent study showed that pre-treatment with rapamycin inhibits AICD in the epithelial cells [38]. In light of these reports, we investigated the effect of low dose rapamycin on AICD in antigen-specific T cells. In order to conveniently obtain an adequate amount of antigen-specific T cells, we harvested splenocytes from DO11.10 mice whose transgenic T cell receptor specifically recognizes OVA. DO11.10 splenocytes were incubated with OVA for 72 hours, and some DO11.10 lymphocytes were treated with 100 nM rapamycin 48 hours prior to OVA stimulation. Cell apoptosis was first assessed using a MitoCapture Detection Kit (BioVision). Disruption of mitochondrial membrane potential is an early event of programmed cell death. In healthy cells, MitoCapture compound aggregates in the mitochondria to produce bright red fluorescence. Alteration of mitochondrial membrane potential in apoptotic cells results in the cytoplasmic accumulation of green fluorescent monomer of the cationic dye. As illustrated in Fig. 6A, a majority of control cells exhibited red fluorescence, whereas OVA stimulation markedly increased green fluorescent apoptotic cells. Conversely, fewer apoptotic cells were detected in the group pretreated with low dose rapamycin. Then, we employed mouse apoptosis real-time PCR array to probe the transcriptional changes of apoptosis-related genes at 24 hours after OVA stimulation. As shown in Fig. 6B, 100 nM rapamycin augmented the transcription of *Bcl2, Bcl2l10, Bnip3l, Mcl1, Nfkb1,* and *Prdx2* in the DO11.10 splenocytes compared to the control group. The up-regulation of these anti-apoptotic genes coincided with less apoptosis in rapamycin-treated cells (Fig. 6A).

**Figure 7:**
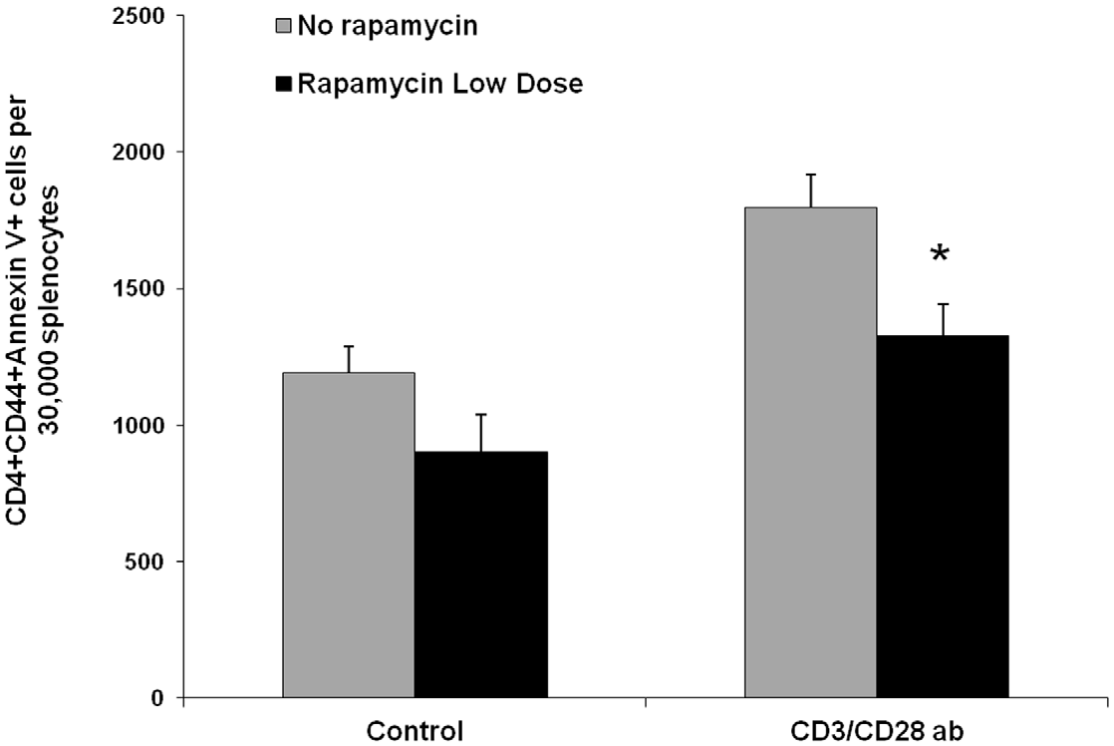
In vivo low dose rapamycin treatment attenuates activation-induced cell death of activated CD4+ T cells. B10.RIII mice were treated with or without daily rapamycin (1.5 µg/mouse) as described previously. On day 21, the spleens were harvested, and further cultured in the presence or absence of anti-CD3 antibody (10 µg/ml) and anti-CD28 antibody (2 µg/ml). Seventy two hours later, the apoptosis of activated CD4+CD44+ T cells was analyzed by flow cytometry for Annexin V staining (All the data represent the mean of 6 animals per group; * p<0.05).

In light of above finding, we asked if low dose rapamycin affected AICD in the EAU model. Thus, B10.RIII mice were immunized with IRBP_161–180_ on day 1, and received intraperitoneal injection of 1.5 µg rapamycin daily. On day 21, the splenocyte were harvested and stimulated with anti-CD3 and CD28 antibodies *in vitro* for 3 days. We used Annexin V staining to specifically examine the apoptosis of CD4+CD44+ T cells. Compared to the control group, activation of T cells by anti-CD3 and anti-CD28 antibodies increased CD4+Annexin V+ lymphocytes (Fig. 7). However, less apoptosis of activated CD4+ T cells was observed in the groups treated with low dose rapamycin *in vivo* (Fig. 7). We also examined apoptotic CD8+ cells in the splenocytes stimulated with anti-CD3 and CD28 antibodies. *In vivo* low dose rapamycin treatment resulted in a slight reduction of apoptosis in activated CD8+ cells, but did not reach a statistical difference as compared to the control group without rapamycin treatment (data not shown). Thus, these data suggest that low dose rapamycin reduces AICD within the responding CD4+ T cell population resulting in a greater number of activated T cells contributing to the autoimmune response.

## Discussion

Generally, B10.RIII mice develop significant EAU after they are immunized with 100 µg IRBP_161–180_ along with pertussis toxin. In this study, to better delineate the difference of ocular inflammation between control and rapamycin-treated groups, we induced EAU with 50 µg/mouse IRBP_161–180_ in the absence of pertussis toxin. With a mild uveitis as a control, we were able to observe the maximal effect of rapamycin on EAU. Control mice developed peak EAU on day 14 in response to the lower dose of IRBP_161–180_, and the uveitis subsided on day 21. However, we have demonstrated that low dose rapamycin exacerbated and prolonged EAU. On day 21, we still observed a robust ocular inflammatory response and retinal damage. Coinciding with this finding, we found that daily treatment of low dose rapamycin increased the frequency of antigen-specific T cells in the mice with EAU. In our unpublished study, we also found that daily treatment of SJL mice with low dose rapamycin consistently augmented PLP_178–191_-induced experimental autoimmune encephalomyelitis. This further validated the effect of low dose rapamycin in an autoimmune response in a different disease setting. To our knowledge, this is the first study showing that low dose rapamycin enhances the autoimmune response in the setting of uveitis. The significance of this observation is twofold.

First, rapamycin is a potent immunosuppressant clinically used to treat transplant rejection and some autoimmune diseases. In light of this finding, suboptimal dosing of rapamycin could exert an immunological effect that is opposite to the desired therapeutic intention. For instance, graft rejection and inflammatory responses may be augmented if an inadequate dose of rapamycin is given to patients. In addition, it is well documented that genetic variations, pharmacokinetics, drug interaction and poor treatment compliance can all affect the serum level of rapamycin [39], [40]. Many patients with organ transplantation or autoimmune diseases commonly receive multiple medications, which can accelerate rapamycin metabolism. Thus, this experiment underscores the need to monitor the pharmacokinetics of rapamycin to ensure the intended therapeutic outcome. In the current clinical trial, it is worth noting that the uveitis patients receive rapamycin via local injection. The local application of rapamycin may achieve better therapeutic concentration in the eye and avoid hepatic biotransformation. Therefore, it would be interesting to compare the efficacy and safety profile between local and systemic therapies of rapamycin in uveitis.

In addition to the potential clinical significance, this study is immunologically intriguing. Although rapamycin is a well documented immune suppressant, we now report that low dose rapamycin can actually exacerbate autoimmune uveitis. This observation is paradoxical to the known effect of rapamycin. However, this finding is in keeping with recent publications suggesting this unexpected impact of low does rapamycin. A number of reports showed the development of interstitial pneumonitis in the absence of infection as well as chronic inflammation-related anemia after rapamycin treatment [11], [12], [41], [42]. Araki et al. have demonstrated that rapamycin promotes virus-specific memory CD8+ T cell immunity in both murine and macaque models [13]. In this study, we found that depletion of CD8+ cells reduced rapamycin-enhanced EAU (Fig. 5C). This suggests that CD8+ lymphocytes contribute to low dose rapamycin-augmented autoimmune response in EAU. In addition, Ford and colleagues report an enhancement of antigen-specific T cell response by rapamycin in the setting of *Listeria monocytogenes* infection [30]. Consistent with this finding, rapamycin has been shown to enhance IL-12 and IL-23 production induced by bacteria [43], [44]. Thus, low dose rapamycin could also augment autoantigen-driven inflammation. It is well documented that IL-12 promotes the development of Th1 cells, whereas IL-23 plays a critical role in Th17 differentiation. Here, we showed that low dose rapamycin augmented IFN-γ- but not IL-17-producing cells (Fig. 4) in the context of EAU. It is unclear if low dose rapamycin treatment skews Th1 differentiation, or it is a phenomenon related to this specific model since IRBP-induced EAU displays a strong Th1 response [45]. Thus, further study is needed.

Recent research in a lymphocytic choriomeningitis virus model has demonstrated that rapamycin treatment during the early T cell expansion phase increases the population of memory T lymphocytes, whereas rapamycin enhances the function of memory cells when they enter the contraction stage [13], [46]. In this study, we found that treating mice with low dose rapamycin during antigen sensitization (days 0–7) led to a severe and sustained EAU. Furthermore, the exacerbation of EAU by low dose rapamycin coincided with increased frequency of the T cells that specifically respond to IRBP. This suggests that low dose rapamycin may augment the T cell response to antigen priming, enhance effector T cell function and contribute to long-term survival. Despite the problematic effect of low dose rapamycin within the setting of autoimmunity, the effect of rapamycin on memory T cells could be beneficial to improve the long-term immune memory for host defense. Whether rapamycin might be used as an effective adjuvant to boost the efficacy of vaccination and immunotherapy against infections or cancers remains to be tested.

Presently, the molecular mechanism by which low dose rapamycin enhances T cell response remains to be fully elucidated. It is well documented that rapamycin inhibits mTOR through the interaction with FK506 binding protein 12 [2]. mTOR is a serine/threonine kinase that plays an important role in regulating the cellular response to external and nutritional cues [46]–[49]. Using RNA-interference knockdown technique, Araki et al. have shown that rapamycin facilitates memory T cell differentiation via mTOR complex 1 pathway [13]. It is thought that metabolic status exerts a pivotal impact on T cell programming. High metabolic state drives effector T cell development, whereas low metabolic cue is in favor of memory T cell differentiation [50]. This notion is further supported by a recent study showing that suppression of fatty acid metabolism promotes a memory T cell response [51]. Thus, inhibition of mTOR by low dose rapamycin may reset the metabolic switch, leading to more memory T cell development. However, high dose rapamycin also blocks mTOR activity. Yet it displays a potent T cell suppression effect. Thus, another mechanism is likely to be involved in the response that develops following low dose rapamycin. After antigen activation, T cells expand rapidly. During the subsequent contraction phase, a majority of effector T cells undergo apoptosis, and a few surviving lymphocytes develop into the memory lineage. A recent study showed that long-term rapamycin treatment can improve mouse longevity [52], suggesting that rapamycin may regulate the apoptosis process. In fact, our study showed that pre-treatment of rapamycin attenuated AICD and apoptotic molecule expression in antigen-activated lymphocytes. In the setting of autoimmune diseases, waves of naïve T cells will gain an access to antigens during different stages of the inflammatory process. Rapamycin-primed naïve lymphocytes could resist AICD after encountering an antigen. Thus, low dose rapamycin may prolong the life span of effector lymphocytes and facilitate the generation of memory T cells by its potential anti-apoptotic effect. However, we believe that inhibition of AICD is only a part of the mechanism by which low dose rapamycin augments the immune response. A recent study demonstrated that autophagy enhances immune response in part by releasing ATP to recruit dendritic cells, CD4+ and CD8+ lymphocytes [53]. Since rapamycin inhibits mTOR, a negative regulator of autophagy [54], [55], low dose rapamycin could exacerbate EAU by augmenting autophagy [56]. Although treatment with rapamycin during different stages of EAU development resulted in a similar outcome of ocular inflammation (Fig. 2A), our study showed that peripheral T cells from the mice treated with low dose rapamycin from day 1 to day 21 exhibited an enhanced IFN-γ response to ocular antigen re-stimulation (Fig. 4). This suggests that low dose rapamycin during other phases of EAU development may augment ocular inflammation through different immune cells and/or mediators.

In summary, our study reveals that low dose rapamycin enhances the autoimmune response in the setting of uveitis. This finding is clinically important as it raises the concern of an unintended consequence from low dose rapamycin and underscores the need to validate dosing and pharmacokinetics during rapamycin therapy. In addition, these data suggest that rapamycin may be used to help enhance a developing immune response, a premise requiring additional investigation in different model systems.
